# Baseline VDJ clonotype detection using a targeted sequencing NGS assay: allowing for subsequent MRD assessment

**DOI:** 10.1038/s41408-020-00343-w

**Published:** 2020-07-22

**Authors:** Malin Hultcrantz, Even Holth Rustad, Venkata Yellapantula, Maria Arcila, Caleb Ho, Mustafa H. Syed, Elli Papaemmanuil, Yanming Zhang, Francesco Maura, Ola Landgren

**Affiliations:** 1 Myeloma Service, Department of Medicine, Memorial Sloan Kettering Cancer Center, New York, NY USA; 2 Hematopathology Laboratory, Department of Pathology, Memorial Sloan Kettering Cancer Center, New York, NY USA; 3 Center for Hematological Malignancies, Department of Medicine, Memorial Sloan Kettering Cancer Center, New York, NY USA; 4 Epidemiology & Biostatistics, Department of Medicine, Memorial Sloan Kettering Cancer Center, New York, NY USA; 5 Cytogenetics Laboratory, Department of Pathology, Memorial Sloan Kettering Cancer Center, New York, NY USA

**Keywords:** Genetics research, Cancer genomics

Dear Editor,

Fluorescent in situ hybridization (FISH), metaphase cytogenetics, and single nucleotide polymorphism (SNP) microarray have long been the standard assays for disease diagnostics and subtyping of multiple myeloma. With the advent of next generation sequencing (NGS), our understanding of the genomic landscape in multiple myeloma has been expanded. Recent data show that NGS-based myeloma-targeted assays can identify immunoglobulin heavy chain (*IGH*) translocations, copy number alterations, as well as somatic mutations^1^. Comprehensive genomic profiling of multiple myeloma is becoming increasingly important for individual risk stratification and therapeutic guidance^2^. Therefore, NGS-based myeloma-targeted assays will gradually replace current standard methods in clinical practice.

In addition to initial genomic profiling of multiple myeloma, the depth of treatment response—measured as minimal residual disease (MRD)—is an important prognostic factor. MRD evaluation with multicolor flow cytometry or immunoglobulin V(D)J sequencing to assess depth of treatment response and to inform clinical decisions is becoming standard in many centers across the USA^3,4^.

Recently, we conducted a head-to-head comparison of an NGS-based multiple myeloma-targeted assay (named “myTYPE”) and found that the targeted assay had a high concordance, >99% sensitivity and specificity, for detection *IGH* translocations and copy number alterations compared to FISH and SNP microarray^1^. In addition, the NGS assay captures somatic mutations in 120 genes that are recurrent and relevant in multiple myeloma^1,5–8^. Here, as a follow-up, we assessed whether the targeted NGS assay can detect patient-specific V(D)J clonotypes at diagnosis, something that is required for subsequent longitudinal MRD tracking.

The commercially available LymphoTrack assays (i.e. *IGH* Leader, FR1, FR2 and FR3; Invivoscribe Inc, San Diego, CA) and our custom capture next NGS-based panel were used to sequence 74 multiple myeloma samples^1,9,10^. Polymerase chain reaction amplification of the *IGH* variable region followed by NGS to a target read depth of >20,000 and clonality calling was performed using LymphoTrack as previously described^10^. The myTYPE assay includes canonical *IGH* locus was included to capture *IGH* (14q32) rearrangements^11^. This locus also includes the V(D)J regions of the *IGH* gene. The MiXCR algorithm was used to reconstruct the V(D)J region from short-read data (Fig. 1)^9,12,13^. The V(D)J clonotypes detected through the targeted NGS panel were then compared to the clonotypes detected in the same samples using the LymphoTrack assay^10^. The study was approved by the Institutional Review Board and all patients were seen at Memorial Sloan Kettering Cancer Center and had consented to the tissue acquisition protocol.

**Fig. 1:**
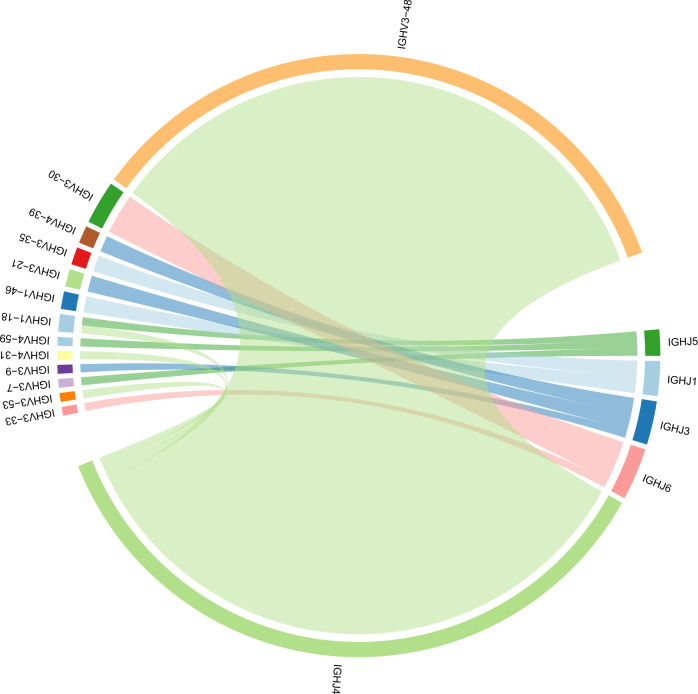
*IGH* translocation in a mutliple myeloma patient. Clonal rearrangement of the immunoglobulin heavy chain V(D)J region in one patient with multiple myeloma reconstructed from targeted panel sequencing of the *IGH* locus.

In 74 samples with a clonal V(D)J sequence detected by Lymphotrack; using myTYPE, the same clonal V(D)J sequence was found in 70 samples (95%). In all 70 samples, the CDR3 sequence detected with myTYPE was identical to the CDR3 sequence reported by LymphoTrack. We went on to examine in detail the four patients where no clonal sequence was detected with myTYPE. There was an adequate sequencing coverage of the *IGH* region in these patients, the median *IGH* coverage ranged between 671 and 1043× in the four samples compared to 842× in the whole cohort. The four samples had bone marrow plasma cell infiltration of 20% or higher and the sample purity ranged between 64 and 84%, estimated through the ASCAT algorithm on the SNP array samples and an in-house algorithm based on the NGS results^14^. One patient had an incomplete V(D)J rearrangement identified by LymphoTrack, without a CDR3 sequence that could be identified by MiXCR. This patient had a hyperdiploid karyotype and a detectable IgG lambda monoclonal protein in serum and no *IGH* translocation detected through FISH or NGS. In the remaining three patients, LymphoTrack identified a productive V(D)J CDR3 sequence as the dominant clonotype. In two of these patients, we could identify the dominant CDR3 sequence in raw sequence data (i.e. FASTQ files) from myTYPE, although it was not called by MiXCR. In the fourth case, the NGS output was successful and the V(D)J clonotype was detected with LymphoTrack but not by with MiXCR or in raw sequence data. Further improvements in algorithm design are expected to increase capture rates close to the current standard of amplifying the entire V(D)J region on a single sequencing read (e.g., LymphoTrack).

In the process of B-cell development, the V(D)J region of the IGH is rearranged to create different combinations of the segments in the variable region of the gene resulting in immunoglobulin with different targets. The large number of variations of the V(D)J sequence and the subsequent somatic hypermutation can however negatively affect the annealing of primers and thus decrease the capture rate. The two available commercial assays have overcome this by including multiple probes primers at various distances from the immunoglobulin variable regions (LymphoTrack) or including probes that cover a wide variety of V(D)J combinations including incomplete rearrangements (ClonoSEQ)^9,15^. Using standard short-read sequencing data, the algorithm MiXCR aligns reads mapping to a library of reference germline V, D, J, and C gene sequences to assemble full-length clonotypes. The algorithm can correct for mismatches and indels that occur during hypermutation^12^. All of the assays routinely used for V(D)J sequencing in the MRD setting require baseline characterization to identify a clonotype for tracking. V(D)J sequencing has an advantage in that the V(D)J clonotypes are stable over time and do not seem to undergo further somatic hypermutation after the diagnosis of multiple myeloma^13^. Thus, DNA sequencing of the V(D)J region is optimal for disease tracking provided that the baseline V(D)J clonotypes are known.

In summary, NGS profiling of multiple myeloma using a myeloma-targeted assay is comparable or better than standard methods, e.g., FISH and SNP microarray, for detecting *IGH* translocations and copy number alterations^1^. Here, we additionally show that by including the *IGH* locus, V(D)J clonotypes can be detected using targeted sequencing. Overall, our findings suggest that baseline genomic profiling–including *IGH* translocations, copy number alterations, recurrent somatic mutations, as well as patient-specific V(D)J clonotypes relevant for longitudinal MRD tracking—can all be captured using a single myeloma-targeted NGS assay.

## References

[1] Yellapantula V (2019). Comprehensive detection of recurring genomic abnormalities: a targeted sequencing approach for multiple myeloma. Blood Cancer J.

[2] Manier S (2017). Genomic complexity of multiple myeloma and its clinical implications. Nat. Rev. Clin. Oncol..

[3] Roshal M (2017). MRD detection in multiple myeloma: comparison between MSKCC 10-color single-tube and EuroFlow 8-color 2-tube methods. Blood Adv..

[4] Mailankody S (2015). Minimal residual disease in multiple myeloma: bringing the bench to the bedside. Nat. Rev. Clin. Oncol..

[5] Bolli N (2018). Analysis of the genomic landscape of multiple myeloma highlights novel prognostic markers and disease subgroups. Leukemia.

[6] Lohr JG (2014). Widespread genetic heterogeneity in multiple myeloma: implications for targeted therapy. Cancer Cell.

[7] Walker BA (2015). Mutational spectrum, copy number changes, and outcome: results of a sequencing study of patients with newly diagnosed myeloma. J Clin Oncol..

[8] Christofferson A (2017). Integrative analysis of the genomic landscape underlying multiple myeloma at diagnosis: an Mmrf commpass analysis. Blood.

[9] Rustad EH (2019). Baseline identification of clonal V(D)J sequences for DNA-based minimal residual disease detection in multiple myeloma. PLoS ONE.

[10] Arcila ME (2019). Establishment of immunoglobulin heavy (IGH) chain clonality testing by next-generation sequencing for routine characterization of B-Cell and plasma cell neoplasms. J. Mol. Diagn..

[11] Walker BA (2013). Characterization of IGH locus breakpoints in multiple myeloma indicates a subset of translocations appear to occur in pregerminal center B cells. Blood.

[12] Bolotin DA (2015). MiXCR: software for comprehensive adaptive immunity profiling. Nat. Methods.

[13] Rustad EH (2019). Stability and uniqueness of clonal immunoglobulin CDR3 sequences for MRD tracking in multiple myeloma. Am. J. Hematol..

[14] Van Loo P (2010). Allele-specific copy number analysis of tumors. Proc. Natl Acad. Sci..

[15] Carlson CS (2013). Using synthetic templates to design an unbiased multiplex PCR assay. Nat. Comm..

